# Optimized Hepatitis E Virus (HEV) Culture and Its Application to Measurements of HEV Infectivity

**DOI:** 10.3390/v12020139

**Published:** 2020-01-24

**Authors:** Nicolas Capelli, Martine Dubois, Mélanie Pucelle, Isabelle Da Silva, Sébastien Lhomme, Florence Abravanel, Sabine Chapuy-Regaud, Jacques Izopet

**Affiliations:** 1 Department of Virology, National Reference Center for HEV, CHU Purpan, 31059 Toulouse, France; 2 Centre de Physiopathologie de Toulouse Purpan (CPTP), Institut National de la Santé et de la Recherche Médicale, Inserm UMR1043, Centre National de la Recherche Scientifique, CNRS UMR5282, Université de Toulouse, 31024 Toulouse, France

**Keywords:** hepatitis E virus, culture, infectivity, TCID50

## Abstract

Hepatitis E virus (HEV) is a major concern in public health worldwide. Infections with HEV genotypes 3, 4, or 7 can lead to chronic hepatitis while genotype 1 infections can trigger severe hepatitis in pregnant women. Infections with all genotypes can worsen chronic liver diseases. As virions are lipid-associated in blood and naked in feces, efficient methods of propagating HEV clinical strains in vitro and evaluating the infectivity of both HEV forms are needed. We evaluated the spread of clinical strains of HEV genotypes 1 (HEV1) and 3 (HEV3) by quantifying viral RNA in culture supernatants and cell lysates. Infectivity was determined by endpoint dilution and calculation of the tissue culture infectious dose 50 (TCID50). An enhanced HEV production could be obtained varying the composition of the medium, including fetal bovine serum (FBS) and dimethylsulfoxide (DMSO) content. This increased TCID50 from 10 to 100-fold and allowed us to quantify HEV1 infectivity. These optimized methods for propagating and measuring HEV infectivity could be applied to health safety processes and will be useful for testing new antiviral drugs.

## 1. Introduction

The hepatitis E virus (HEV) is one of the most common causes of hepatitis worldwide. HEV is classified in the *Orthohepevirus A* species that includes at least eight genotypes, five of which (HEV1 to HEV4 and HEV7) infect humans [1,2]. HEV1 and 2 are restricted to humans; their transmission is linked to poor sanitation and contaminated water. HEV3 and 4 are prevalent in industrialized countries where they are zoonotic; their reservoir includes pigs, wild boar, deer, and rabbits [3,4,5,6]. HEV7 was identified in camel and was found in an immunocompromised patient [7]. HEV can also be transmitted by blood transfusion [8].

HEV causes mainly self-limiting acute hepatitis, but severe hepatitis in pregnant women infected by HEV1 [9] and chronic hepatitis in immunocompromised patients infected by HEV3, 4, and 7 can occur [10]. HEV1–4 can worsen chronic liver disorders and can be associated with a range of extra-hepatic manifestations [7,10,11]. While ribavirin therapy efficiently overcomes most cases of chronic hepatitis E [12], cases of ribavirin failure have been reported, indicating that additional drugs should be developed [13].

The 7.2 kb-long single-stranded positive-sense RNA genome of HEV encodes three open reading frames (ORFs) flanked by 5′ and 3′ untranslated regions [14]. ORF1 is a non-structural protein, including seven functional domains: methyltransferase domain (MeT), Y domain, papain-like cysteine protease (PCP), hypervariable region (HVR or polyproline region), X domain (Macro domain), helicase domain, and RNA-dependent RNA polymerase (RdRp) [10]. Insertions in the HVR can confer advantages for replication [15,16]. ORF2 is translated from two initiation codons giving two forms of ORF2, the capsid protein, and a soluble glycosylated protein [17,18], while ORF3 is a small phosphorylated and palmitoylated protein involved in HEV morphogenesis and release [19,20,21,22,23]. The HEV particles that leave infected cells are in a quasi-enveloped form (eHEV) [24]. This implies that HEV uses cell membranes to bud in a lipid-associated form that protects the particle from neutralizing antibodies [25,26]. However, these HEV particles seem to get rid of their lipids in the digestive tract, and leave the host in a naked form (nHEV) [27].

Genotype 3 HEV has been cultured on several hepatocarcinoma cell lines, including PLC/PRF/5 and HepG2/C3A, as well as on A549 lung carcinoma cells [28]. Different culture conditions have been used, varying the percentage of fetal bovine serum (FBS) in the culture medium, the additional components, such as MgCl_2_ or dimethylsulfoxide (DMSO), the time of inoculation, and the temperature of the culture [25,29,30]. Recently, we isolated a subclone of the HepG2/C3A cell line, called HepG2/F2, which mimics the main physiological characteristics of hepatocytes when grown in a DMSO-containing medium, with an apical side corresponding to bile and a basolateral side corresponding to blood. We used this system to analyze the characteristics of HEV particles on the two sides and demonstrated that HEV is secreted in a lipid-associated form at both sides [30].

We have also developed an endpoint-dilution method for calculating tissue culture infectious dose 50 (TCID50) that is based on HEV culture and quantifying HEV RNA. This showed that naked particles from stools were more infective than lipid-associated particles from culture supernatants [26]. TCID50 has also been used to compare the infectivity of HEV3 strains on PLC/PRF/5 cells [31].

The work described here compares several culture conditions for HEV3. An enhanced HEV production could be obtained varying the composition of the medium, including FBS and DMSO content. These conditions could be applied to the growth of a HEV1 strain that does not efficiently grow in previous conditions. These culture conditions were then used to improve the quantification of HEV infectivity.

## 2. Materials and Methods

### 2.1. Reagents

Dulbecco’s modified Eagle’s medium (DMEM), Medium 199 (M199), William’s E medium (32551), fetal bovine serum (FBS), 0.05% trypsin-EDTA containing phenol red, phosphate-buffered saline without calcium and magnesium (PBS), were all purchased from Thermo Fisher Scientific, Life Technologies SAS (Saint-Aubin, France). FBS was heat-inactivated and, when mentioned, it was depleted of exosomes by ultracentrifugation (110,000 g; 14 h; 4 °C), and stored at −20 °C before used. Penicillin G (10,000 U/mL), streptomycin (10,000 U/mL), and amphotericin B (25 µg/mL) were supplied as a 100X mixture (PSA) by Bio Whittaker-Lonza (Amboise, France). DMSO was from Sigma-Aldrich (Saint-Quentin-Fallavier, France).

### 2.2. HEV Strains

#### 2.2.1. nHEV

We used clinical strains from patients’ stools at the acute phase of the infection: a genotype 3f strain (TLS09-0, GenBank accession number KC166967) [16] and a genotype 1 strain (VIC-G1, GenBank accession number MN401238) [32]. These were prepared and stored at −80 °C as previously described [26].

#### 2.2.2. eHEV

These samples of nHEV were used to infect PLC/PRF/5 cells (ATCC^®^ CRL-8024™) and samples of supernatant were taken on days 15 to 40 post-infection, centrifuged at 300 g, and frozen at −80 °C in a single bottle. The pooled samples were thawed and concentrated on an Amicon^®^ Ultra Centrifugal Filter Unit (100 kD cut-off, Millipore, Molsheim, France). Aliquots were frozen at −80 °C.

### 2.3. Infection and Culture

HepG2/C3A cells (ATCC^®^ CRL-10741TM) or the HepG2/C3A subclone F2 [30] were grown in DMEM supplemented with 10% heat-inactivated FBS at 37 °C in a 5% CO_2_ humidified atmosphere. Cells for infection were detached with trypsin-EDTA and distributed in 12-well plates (4.10^5^ cell per well) or 96-well plates (10^5^ cells per well). Two days later, when they were confluent, the cells were washed once with PBS. An aliquot of virus suspension was diluted in one of the following media: MCCI (47% DMEM, 47% M199, 3% MgCl_2_ 1 M, 2% heat-inactivated FBS, 1% PSA), WED (96% William’s medium E, 1% DMSO, 2% heat-inactivated exosome-free FBS, 1% PSA) or DSD (87% DMEM, 2% DMSO, 10% heat-inactivated FBS, 1% PSA) and added to each well. The plates were incubated for one hour at room temperature or 6 h at 35.5 °C. The inoculum was then removed, culture medium was added, and the plate incubated overnight at 35.5 °C in a 5% CO_2_ humidified atmosphere. The cells were then washed five times with PBS and grown in the indicated culture medium. Aliquots (1.5 mL) of supernatant were collected every two days and replaced with the same volume of pre-warmed culture medium.

### 2.4. HEV RNA Quantification

HEV RNA was extracted from samples (140 µL) using the QiaAmp viral RNA mini kit (Qiagen, Courtaboeuf, France) and quantified by RT-PCR of the ORF3 gene [33]. This method is accredited ISO 15189 and has a limit of detection of 80 HEV RNA copies/mL [34]. It can be used to detect and quantify HEV1 to HEV4 RNA [35,36].

### 2.5. Determination of the Tissue Culture Infectious Dose 50 (TCID50)

TCID50/10^6^ HEV RNA copies were determined as previously described [26]. Briefly, tenfold dilutions of the virus suspension were used to infect HepG2/C3A cells or the F2 clone in 96-well plates (10^5^ cells in 300 µL per well) as described above. Each dilution was tested on six replicates in each experiment. HEV RNA in cell lysates was quantified at day 10 post-infection and the TCID50 obtained using the calculations of Reed and Muench [37].

### 2.6. Statistical Analysis

The Mann–Whitney U test was used to compare quantitative variables when the replicate number was ≥4, using GraphPad Prism 7 software (version 7.03, GraphPad Software, La Jolla, USA). The *p* values of <0.05 were considered statistically significant. The overlap of ranges was also considered [38].

## 3. Results

### 3.1. Enhanced HEV Cultures

**Figure 1:**
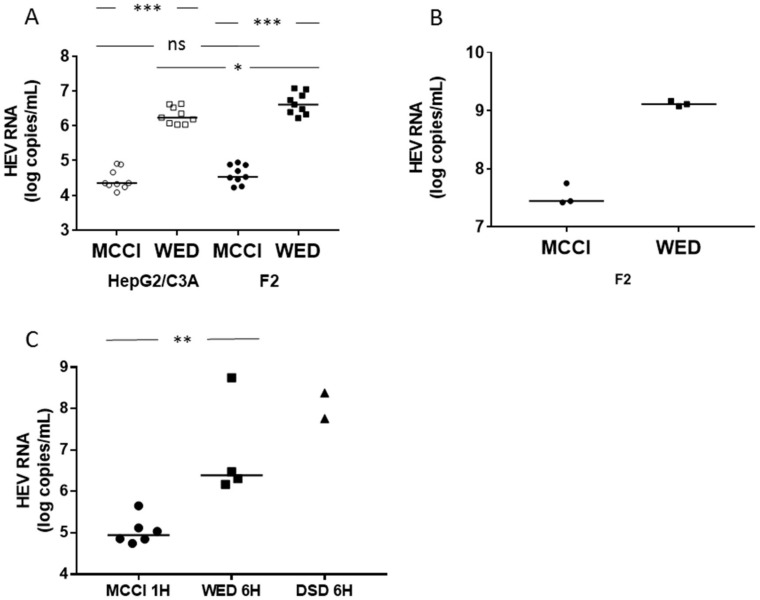
Hepatitis E virus genotype 3 (HEV3) RNA production in different culture conditions. (**A**) HepG2/C3A (empty symbols) or HepG2/F2 (filled symbols) cells were seeded on 24-well plates at 4.10^5^ cells/wells and infected with the same suspension of eHEV3, at 473 HEV RNA copies/cell, diluted in MCCI (circles) or WED (squares) medium, for one hour at 35.5 °C. Cells were maintained in the same medium for 15 days, at which time the HEV RNA in the culture supernatant was quantified. *n* = 9 in all conditions. The horizontal bars represent medians. * = *p* < 0.05. *** = *p* < 0.001. (**B**) HepG2/F2 cells were seeded and infected as in (A) and cultured for 36 days. They were then washed and lysed by freezing/thawing and the HEV RNA in the lysate was assayed. The horizontal bars represent medians and *n* = 3 in both conditions. (**C**) HepG2/C3A cells were seeded at a density of 10^5^ cells/well of a 96-well plate and infected with the same suspension of eHEV3 (100 HEV RNA copies/cell) diluted in MCCI for one hour at 35.5 °C (circles, *n* = 6), or in WED (squares, *n* = 4) or DSD (triangles, *n* = 2) medium for 6 h at 35.5 °C. Cells were maintained with the same medium for 10 days, at which time the HEV RNA in the cell lysate was quantified. The horizontal bars represent medians. ** = *p* < 0.01.

We inoculated HepG2/C3A or HepG2/F2 cells with an eHEV3 sample (473 HEV RNA copies/cell) in DMEM/M199 medium containing 2% FBS and 30 mM MgCl_2_ (MCCI) [25], or in William’s E medium containing 2% FBS and 1 % DMSO (WED) for one hour at 35.5 °C [30]. The HEV RNA in the culture supernatant was quantified by RT-PCR on day 15 post-infection. The HEV RNA in HepG2/C3A cells grown in MCCI reached 4.35 [range: 4.09–4.91] log HEV RNA copies/mL, while it reached 6.24 [range: 6.03–6.63] in the same cells grown in WED (*n* = 9, *p* < 0.001). The HEV RNA concentration in HepG2/F2 cells grown in MCCI was 4.53 [range: 4.23–4.95] log HEV RNA copies/mL, while it was 6.61 [range: 6.22–7.08] in these same cells grown in WED (*n* = 9, *p* < 0.001) (Figure 1A). This difference was also found within cells. We measured the HEV RNA concentration in F2 cell lysates at the end of the culture (day 36 post-infection) (pi). It was 7.44 [range: 7.42–7.75] log copies of HEV RNA/mL in cells grown on MCCI and 9.11 [range: 9.08–9.16] in cells grown on WED (Figure 1B). The same results showed HEV RNA concentration was significantly higher in HepG2/F2 cells than in the HepG2/C3A mother line (*n* = 9, *p* < 0.05) when the cells were grown in WED medium (Figure 1A).

Yin et al. found that the optimum incubation time of the inoculum in DSD medium was 6 h [29]. We measured the HEV RNA concentration in cells cultured in WED and DSD after incubation with eHEV3 (100 HEV RNA copies/cell) inoculum for 6 h and under our basal conditions: culture in MCCI after incubation with the same inoculum for 1 h. HEV RNA in the cell lysate was quantified on day 10 post-infection. It was 4.95 [range: 4.74–5.65, *n* = 6] log copies of HEV RNA/mL in cells incubated in MCCI, 6.39 [range: 6.17–8.75, *n* = 4] in cells grown in WED, and 8.07 [7.76 and 8.38, *n* = 2], in cells cultured in DSD. The difference between cells grown in MCCI and WED was significant (*p* < 0.01) without overlap of the values, while the values in DSD and WED were in the same range (Figure 1C).

These results indicate that culturing cells in WED, after inoculation for one or 6 h, or in DSD, after a 6-h inoculation, increased HEV RNA production 10-fold or more over that obtained from cells cultured in MCCI after one-hour inoculation. This increase cannot be explained by an increased cell proliferation because, in the presence of DMSO, these cells form a cell monolayer when they reach confluence at day 3 or 4 and stop growing [30,39].

### 3.2. Impact of Culture Conditions on TCID50

In WED conditions, HEV3 RNA production in HepG2/F2 subclone cells was better than in HepG2/C3A cells (Figure 1A and [30]). So, we compared the TCID50 data for these cell lines. The difference was not significant (data not shown), but the F2 cells were more readily detached by the PBS washes, hampering cell manipulations. We therefore used HepG2/C3A cells in subsequent experiments.

We determined the TCID50 of the HEV3 strain using WED and DSD media with a 6-h inoculum incubation, and compared the results to those obtained using MCCI with a one-hour inoculum incubation. HepG2/C3A cells were inoculated with successive ten-fold dilutions of nHEV3 (stool sample) or eHEV3 (first-passage supernatant culture on PLC/PRF/5) (6 replicates). HEV RNA was quantified on day 10 post-infection and TCID50s per 10^6^ HEV RNA copies in the inoculum were calculated.

**Figure 2:**
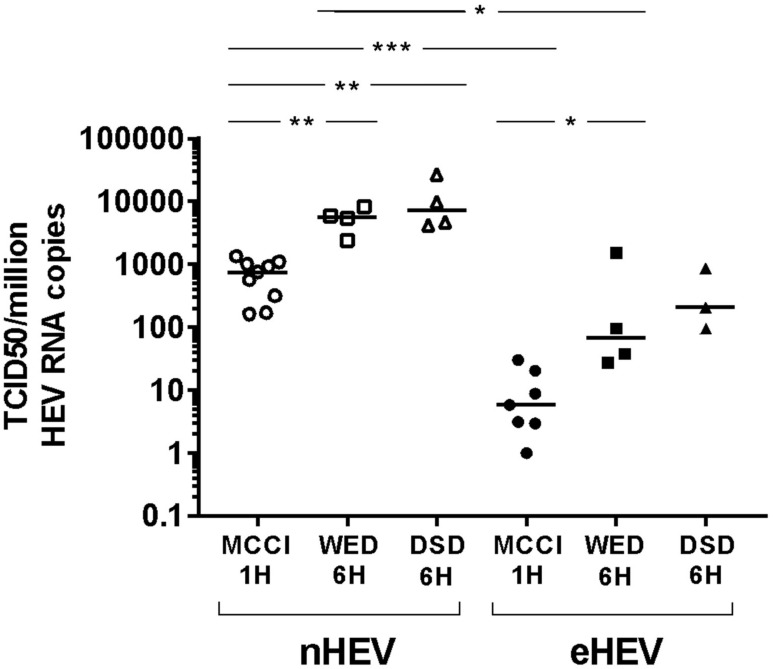
HEV3 RNA infectivity in different culture conditions. Tissue culture infectious dose 50 (TCID50) per million HEV RNA copies calculated for nHEV3 (empty symbols) and from the first-passage supernatant culture of the same strain on PLC/PRF/5 cells, eHEV3 (filled symbols). Successive ten-fold dilutions of the viral suspensions were used to inoculate HepG2/C3A cells, either during 1-h at room temperature in MCCI medium (circles) or for 6 h at 35.5 °C in WED (squares) or DSD (triangles) medium. Cells were maintained in the same medium until day 10. The horizontal bars represent medians with range. * *p* < 0.05. ** *p* < 0.01. *** *p* < 0.001.

The TCID50/10^6^ HEV RNA copies of nHEV was 750.3 [range: 160.8–1334, *n* = 9] in MCCI conditions, 5590 [range: 2383–8294, *n* = 4] in WED, and 7200 [Min: 4214–Max: 27060, *n* = 4] in DSD conditions. The TCID50/10^6^ HEV RNA copies of eHEV was 5.9 [Min: 1.0–Max: 30, *n* = 7] in MCCI conditions, 67 [range: 28–1544, *n* = 4] in WED, and 207 [Min: 97–Max: 875, *n* = 4] in DSD conditions (Figure 2).

The difference between MCCI with a one-hour inoculum incubation and WED with a 6-h inoculum incubation, or MCCI with a one-hour inoculum incubation and DSD with a 6-h inoculum incubation, for both nHEV and eHEV, was attested by *p* values <0.05 and by only two overlapping values between MCCI and WED. In contrast, there was no difference between the WED and DSD conditions (*p* > 0.05 and large range overlaps) (Figure 2).

Thus, the ranges of nHEV and eHEV values did not overlap when cells were cultured in the same conditions, and nHEV was more infective than eHEV (*p* < 0.05 for MCCI or WED conditions) (Figure 2).

### 3.3. Culture and Infectivity of a Genotype 1 Strain

**Figure 3:**
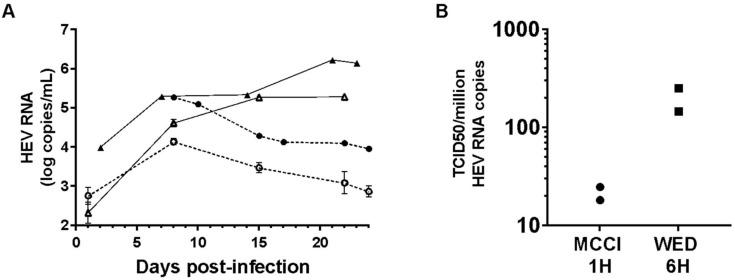
HEV1 culture and infectivity. (**A**) HepG2/C3A cells were infected with nHEV1 in MCCI at 25 (empty circles) or 50 (filled circles) HEV RNA copies/cell for one hour (dashed line), or in DSD at 15 (empty triangles) or 50 (filled triangles) HEV RNA copies/cell for 6 h (solid line). The graph shows the HEV RNA in the culture supernatant as a function of time (error bars: standard deviations). (**B**) TCID50/10^6^ HEV RNA copies of nHEV1 (*n* = 2) after inoculation of HepG2/C3A cells with virus suspension in MCCI (circles) for 1-h on or with virus in WED (squares) for 6 h, each followed by cell culture in the inoculum medium.

We used the improved HEV3 culture conditions to grow a HEV1 clinical strain. HepG2/C3A cells were inoculated with nHEV1 in MCCI for one hour or in DSD for 6 h. After 8 days in MCCI, HEV RNA in the culture supernatant reached 4.14 log HEV RNA copies/mL (inoculum of 25 HEV RNA copies/cell) and 5.27 (50 HEV RNA copies/cell) and then decreased over the following two weeks. The HEV RNA released by cells cultured in DSD increased from day 2 post-infection and plateaued at 5–6 log copies/mL until the end of the experiment (24 days post-infection) (Figure 3A).

Next, we measured the infectivity of nHEV1. The TCID50/10^6^ HEV RNA copies from two independent experiments were 18 and 24 in MCCI conditions and 147 and 250 in WED conditions (Figure 3B).

## 4. Discussion

Our comparison of HEV culture conditions allowed us to optimize the assay of HEV infectivity. This assay combined an optimized culture system and a highly sensitive method for quantification of HEV RNA.

The infectivity of a virus can be estimated using the physical properties of its particles by transmission electron microscopy but this cannot be used in routine experiments. Flow virometry can sort virus particles according to their main characteristics (size, genome content, surface protein content, lipid composition), which can then be linked to their infectivity, but this method is not yet fully developed [40]. Culture-based methods must combine efficient culture and detection systems. Measurement of Fluorescence Focus Units (FFU) has been used for HEV [15] but it assumes that a good antibody is available for detecting infection. In contrast, HEV RNA assays are easily standardized and highly sensitive, detecting as few as 80 copies/mL of HEV RNA (corresponding to 60 IU/mL) [34]. It is important to ensure that the inoculum is removed before the RNA is assayed. We have checked that the HEV RNA detected in PBS-washed cells on day 4 post-inoculation is not a residue of the input virus [30].

We optimized the performance of the measurements of HEV infectivity by enhancing HEV culture efficiency. We find that both the WED and DSD media increase intracellular and extracellular HEV RNA production compared to MCCI. Several parameters could affect this HEV culture efficiency. Among them, exosome-depleted FBS was added to WED while non-depleted FBS was added to MCCI. Exosome-depletion of FBS alters cell growth [41,42] but in T-lymphocytic cell lines, it promotes their infection by the Human Immunodeficiency Virus [42]. Concerning HEV, we previously checked that adding depleted-exosomes FBS to MCCI did not alter HEV RNA production compared to non-depleted FBS (unpublished data). DSD contained 10% FBS, while MCCI 2%. The production of ORF2 antigen was shown to follow a FBS dose-effect but the effect on HEV RNA production was not evaluated [31]. We did not evidence differences between WED containing 2% of exosome-depleted FBS and DSD containing 10% non-depleted FBS on HEV RNA production. Further investigations will be necessary to elucidate the relative impact of FBS concentration and FBS exosome-depletion on HEV infection.

The common feature between WED and DSD is that they contain DMSO, which promotes hepatocyte differentiation. DMSO also led to the expression of liver-specific functions in cells isolated from a patient’s liver tumor, rendering them sensitive to infection by the Hepatitis B virus (HepaRG cell line) [43]. DMSO modifies the metabolic activity of Huh-7 tumor cells to give a phenotype similar to that of primary hepatocytes by increasing the secretion of albumin or alpha-1 antitrypsin; this stimulates the replication of strain JFH-1 of the Hepatitis C virus (HCV) in vitro [44]. Several HepG2 clones were polarized using 1% DMSO to study the vectorial release of HCV [45]. The HepG2/ F2 cells develop a polarized phenotype in a medium containing 1% DMSO and support the growth of HEV3 and HEV1 strains [30]. The increased HEV RNA production observed in HepG2/F2 compared to HepG2/C3A could be related to the better ability of the HepG2/F2 cells to polarize. The link between polarization and sensitivity to HEV infection still needs to be investigated.

The inoculum incubation time is also an important factor in HEV culture. Yin et al. showed that 6 h for eHEV and 4 h for nHEV are needed for the maximal entry of HEV particles in cells [29]. Although we could not evaluate a 6-h inoculum incubation time in MCCI, an increase of the same order of magnitude was observed with one-hour or 6-h inoculum incubation times in WED compared to a one-hour incubation time in MCCI. This suggests that the medium composition has a higher impact on HEV production than the inoculum incubation time.

Further work is ongoing to decipher the relative role of FBS, DMSO, cell line, and incubation time on HEV spreading. However, at this point, WED or DSD conditions, with a 6-h inoculum incubation time, allow us to grow and to measure infectivity of HEV strains that could not be measured in MCCI conditions with a one-hour incubation time.

We find that the culture supernatant of HEV-infected PLC/PRF/5 cells contain ten to a hundred times fewer infectious particles per million HEV RNA copies than do feces. This difference in infectivity has also been shown for the culture supernatant of HEV-infected HepG2/C3A cells [15,26]. It has been linked to the use of distinct entry mechanisms, with nHEV and eHEV having different binding kinetics and eHEV requiring endocytic delipidation [29].

Few human clinical isolates of HEV1 have been cultivated (e.g., Sar-55, 87A, F23) on PLC/PRF/5 or HepG2/C3A hepatoma cells [28,46] and most of the data on the life cycle of HEV1 were obtained using the cDNA clone of Sar-55 [47]. Recently, HEV1 strains have grown on primary decidua or placenta cells [48], or on induced pluripotent stem cell-derived hepatocyte-like cells [49], but these cells are not readily available and their use requires particular skills. Our results indicate that the HEV RNA produced in HepG2/C3A cultures infected with a clinical strain of HEV1 from stools and its TCID50 values can be greatly increased by using a DMSO-containing medium.

Our system will quantify the infectivity of naked or quasi-enveloped forms of strains isolated from different biological compartments, as early as after just one passage in culture. We applied it to a urine sample whose infectivity could not be assayed in MCCI but was possible in DSD [50]. In addition, this method enables us to measure the infectivity of particles released from the basolateral side of polarized HepG2/F2 cells grown on inserts, despite the fact that they contained low concentrations of HEV RNA [30]. These optimized methods for HEV propagation and infectivity measurement will be useful for evaluating the impact of health safety processes on HEV and for assessing new antiviral drugs.
